# Clinical outcomes of giant cell tumor of bone treated with bone cement filling and internal fixation, and oral bisphosphonates

**DOI:** 10.3892/ol.2012.1036

**Published:** 2012-11-20

**Authors:** XIUCHUN YU, MING XU, SONGFENG XU, QING SU

**Affiliations:** Orthopedic Department, General Hospital of Jinan Military Commanding Region, Jinan, Shandong 250031, P.R. China

**Keywords:** giant cell tumor, cement filling, internal fixation, bisphosphonate

## Abstract

Giant cell tumor (GCT) of the bone is a relatively common primary bone tumor. Treatment with simple curettage often results in a high local recurrence rate. Tumor resection and reconstruction with prosthesis or an allograft has a low rate of local recurrence; however, the patient’s native joint function becomes significantly impaired. With the development and usage of aggressive curettage, it is a priority to treat GCT with a method that reduces the local recurrence rate and preserves the native joint. To evaluate the feasibility of treating GCT with aggressive curettage and cement filling using internal fixation and oral bisphosphonates, 16 patients with GCT of the bone located in the distal femur and treated in our department from January 2008 to June 2011, were followed up. The patients had received aggressive curettage, bone cement filling, internal fixation and oral administration of bisphosphonates.There were seven males and nine females in total, with a mean age of 38 years. All patients were carefully assessed prior to surgery in order to determine the integrity of the tumor cavity. Subsequently, patients were treated with aggressive curettage by high-speed burring and cementation with internal fixation, and were administered postoperative oral alendronate sodium tablets (10 mg/day) for two years. The median follow-up time was 25 months. None of the patients were lost to follow-up. No local recurrence or metastasis was observed in the last follow-up. The Enneking limb function score range of the affected limb was 24–29 (average, 26.7). At the last follow-up, all patients exhibited solid fixation without fracture of the subchondral bone in plain radiographs. Based on these data, we suggest that patients with distal femoral GCT may be treated with aggressive curettage and cement filling, with internal fixation and oral bisphosphonates. The advantages of this method are its safety and efficacy. However, the long-term outcomes require further investigation.

## Introduction

Giant cell tumor (GCT) of the bone is a relatively common primary bone tumor, accounting for 5% of all invasive primary bone tumors. Treatment with simple curettage often results in a high local recurrence rate. Tumor resection and reconstruction with prosthesis or a large segment allograft has a low rate of local recurrence; however, the patient’s native joint function becomes significantly impaired. With the development of surgical techniques and an increased understanding of the biological behaviour of GCT, curettage has gradually been replaced by aggressive curettage (1). This implies using high-speed burr to grind the paratumorous bone, expanding the range of curettage and using chemical agents (phenol, alcohol and bone cement) to process the bone cavity, to finally achieve marginal excision. We followed up 16 patients with GCT of the bone who had been treated in the Orthopedic Department of the General Hospital of Jinan Military Commanding Region, China, from January 2008 to June 2011. These patients had received aggressive curettage, bone cement filling, internal fixation and oral administration of bisphosphonates. In the follow-up, tumor recurrence and joint function were observed in order to evaluate the clinical outcomes.

## Patients and methods

### Patients

Among the 16 patients with GCT in the distal femur that were treated in our hospital from January 2008 to June 2011, there were seven males and nine females, aged 27–78 years (mean, 38 years). There were 12 cases of primary GCT and four cases of recurrent GCT. This study was approved by the ethics committee of the Orthopedic Department, The General Hospital of Jinan Militray Commanding Region, Jinan, Shandong, China. Informed consent was obtained from the patients.

### Clinical diagnosis and classification

Patients were evaluated with clinical and imaging data following examination with X-ray, CT and MRI. Preoperative biopsy was conducted on the 12 patients with primary GCT, in order to confirm the diagnosis. According to the Campanacci grading system (2); two cases of grade I, 11 cases of grade II and three cases of grade III were present in this group.

### Surgical technique and adjuvant therapy

**Figure 1.**
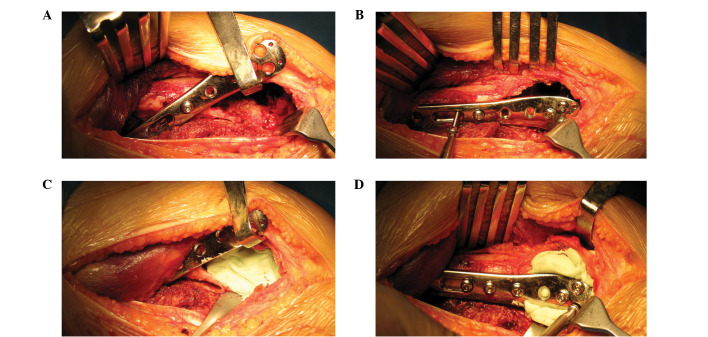
The bone cement filling and plate fixation process. (A) Treating the tumor wall with a scraper, a high-speed burr and an electric knife, and selecting the appropriate length temporary screws; (B) threading a screw into the temporary, steel fixation plate and lifting the plate; (C) filling the bone with cement; (D) rapidly tightening the screw along the nail rod.

The surgical approach was determined based on the location of the lesion. The tumor was exposed completely to separate the normal adjacent soft tissues. While protecting the adjacent soft tissues, a bone window was opened as wide as possible from the lesion edge. The tumor was then removed with a scraper, the curettage was further expanded with a high-speed burr and the paratumorous cortical bone was ground to form smooth surface. The spongy bone was ground 0.5–1.0 cm and the surface of exposed cortical bone was burned using an electric knife. The lesion was then washed with a large volume of distilled water under pulse pressure. An appropriate anatomical steel plate was selected and the healthy diaphysis, as well as the ground paratumorous cortical bone, were drilled and tapped. Screws of the appropriate length were selected and screwed in at the correct entry angle. All screws were then removed, apart from one screw that served to temporarily fix the steel plate, which was then lifted to fill the bone with cement. After bone cement filling, all screws were immediately tightened along the original threads (Fig. 1).

All patients were administered postoperative, oral alendronate sodium tablets (10 mg/day); drug administration was pulsed for one month every two months, and this continued for two years.

### Follow-up

Following surgery, all patients were followed up regularly. The follow-up time was 23–53 months (median, 28 months). The follow-up review included the following: Lung CT scan once every six months and X-ray examination once every three months for two years, and then every six months after the first two years. Limb function was simultaneously evaluated by Enneking limb reconstruction scores (3).

## Results

### Oncology results

All patients were followed up. The median follow-up time was 28 months (23–53 months) and no local recurrence or metastasis was observed.

### Limb function

**Figure 2.**
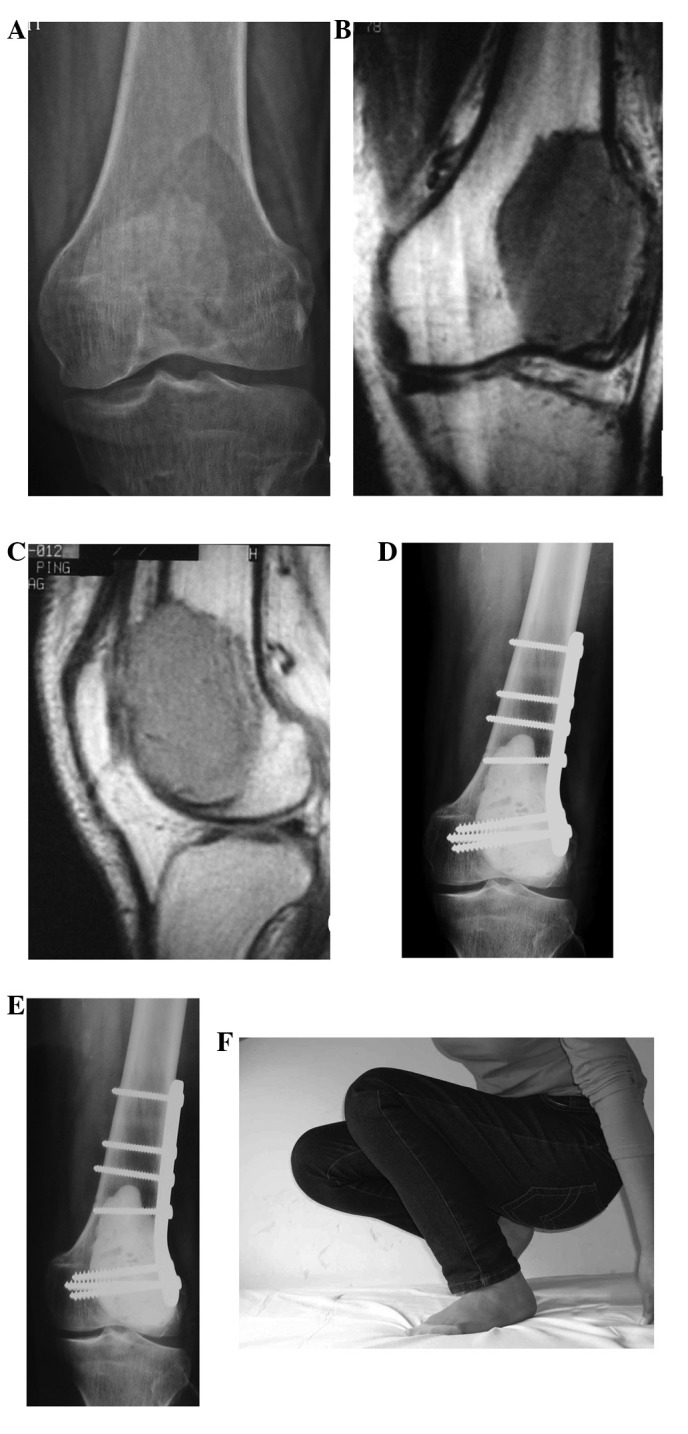
A 30-year-old female patient with giant cell tumor (GCT) of the left distal femur. (A) X-ray film reveals osteolytic destruction and cortical thinning at the left femoral condyle. (B) and (C) T1-weighted MRI reveals low signal at the distal femur and a lateral visible tumor penetrating the front side of the cortex. (D) 4 days after surgery, postoperative X-ray reveals bone cement filling and internal fixation are normal. (E) and (F) 22 months after surgery, X-ray shows that the joint space is normal, no lucent zones surround the bone cement, internal fixations are firm and the joint function is normal.

All incisions healed well. The postoperative training of the knee joint was initiated three to five days after surgery and the weight-bearing exercise began 14 days after surgery. Limb function had essentially recovered after one month. Only one patient suffered from pain when bending the knee, as a screw was too long. Repeat surgery was carried out to remove the screw, and the postoperative knee function returned to normal. Enneking limb function scores of this group of patients ranged from 24–29 (average, 26.7) (Fig. 2).

### Imaging results

**Figure 3.**
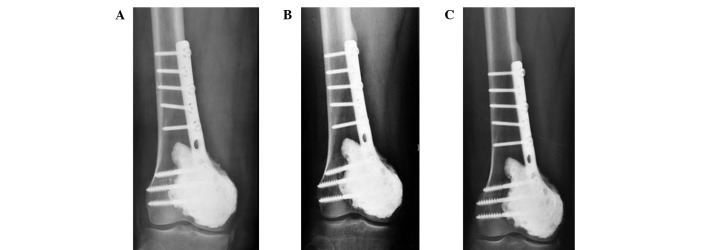
A 41-year-old male patient with GCT of the right distal femur. (A) 4 days after surgery, X-ray film reveals bone cement filling and internal fixation are good. (B) 12 months after surgery, X-ray film shows the bone cement surrounding the lucent zones, normal joint space and internal fixation. (C) 31 months after surgery, the bone cement surrounds lucent zone with no progression.

All patients received X-ray examination once every three months within the first two years after surgery, and then once every six months after two years. All internal fixations remained in place in this group of patients and no subchondral bone fracture was identified. Four patients were observed to have lucent zones evenly distributed around the bone cement during 4–13 months (average, 8 months) of follow-up (Fig. 3). However, these lucent zones demonstrated no further progression in further follow-up.

### Adverse drug reaction

Two patients suffered from acid reflux, heartburn and other mild gastrointestinal symptoms after oral alendronate, which disappeared following treatment of these symptoms.

## Discussion

GCT is a relatively common primary bone tumor. Although GCT is benign, it is typically clinically treated with intralesional curettage and en bloc resection, due to its strong local invasion and high postoperative recurrence rate. Simple intralesional surgery has a relatively high recurrence rate. As studies have demonstrated, the recurrence rate for GCT treated by intralesional curettage plus bone grafting was 29–75% (4). Although en bloc resection has a lower recurrence rate, it damages the patient’s joint function and requires reconstruction with a large segment allograft or prosthesis. In the case of long-term survival, the emergence of various complications becomes inevitable; therefore, the long-term outcomes of en bloc resection are poor.

With the gradually increasing awareness of the local invasion of GCT and the development of surgical techniques, the concept of aggressive curettage has arisen. This implies that the affected bone and wall are ground intralesionally with a high-speed burr, washed under pulse pressure and treated with chemical agents (including phenol, alcohol and bone cement) to achieve marginal excision. Algawahmed *et al*(5) used meta-analysis to analyze 13 articles regarding the intralesional application of a high-speed burr in GCT, with or without auxiliary treatments, and identified: i) 66 recurrences (20%) out of 323 cases that had received treatment with both a high-speed burr and another auxiliary method, and ii) 15 recurrences (23%) out of 64 patients who had received simple treatment with a high-speed burr only. The authors proposed that using a high-speed burr is the most effective way to reduce the local recurrence rate. Treatment of GCT with aggressive curettage retains the limb function and reduces the recurrence rate to a minimum. However, there remains a lack of uniform surgical recommendation regarding aggressive curettage. We propose that a comprehensive preoperative assessment of the biological behavior of GCT, including tumor growth, clinical progression, as well as pathological and imaging manifestations, should be conducted. Aggressive curettage may be performed in limb GCT cases without pathological fracture, with limited tumor location and extent. It is not necessarily a contraindication to use intralesional curettage for patients with Campanacci grade III tumors.

Limb GCT often occurs around the knee, and most patients have a long-term survival, which results in a reasonably high demand on the limb function. Intralesional curettage with bone grafting may require a large amount of bone graft, and it is difficult to differentiate the bone graft absorption from recurrence. Moreover, the required protective weight-bearing before bone healing interferes with the recovery of joint function. Bone cement as a filler material has long been used in GCT lesion filling and exhibits numerous advantages. It reconstructs bone defects immediately, restores bone continuity and facilitates early postoperative weight-bearing. In a biomechanical study by Frassica *et al*(6), with the application of bone cement reconstruction, the mechanical strength of bone defects was demonstrated to be restored to 98%. Additionally, the heat released by bone cement polymerization exerts a high-temperature inactivation effect on the lesion edge and further reduces the recurrence rate. In a follow-up study by Kivioja *et al*(7), the recurrence rate of 147 patients with intra-lesional curettage and bone cement filling was 22%, while that of 47 cases receiving curettage and bone grafting was 52%. Moreover, Kafchitsas *et al*(8) performed curettage on 38 GCT patients, among which 21 patients who had received bone cement filling demonstrated a recurrence rate of 23.8%, while the other 17 patients who had received bone grafting displayed a postoperative recurrence rate of 52.9%. Bone cement filling is also beneficial as it enables tumor recurrence to be detected early-on in X-ray film. Kafchitsas *et al*(8) followed up 21 patients with bone cement filling by imaging, and revealed that a gradually progressive lucent zone between the bone and bone cement was present in four out of the five recurrent cases. The authors propose that this phenomenon may be used as a reliable indicator of tumor recurrence. During the follow-up, they also observed that patients who had received bone cement filling demonstrated a 1.4 mm (average) lucent zone with a hardened edge between the bone and bone cement in the first six months after surgery. However, the lucent zones typically did not progress and affect the fixation. Furthermore, the width of the lucent zone was related to the volume of bone cement; the authors hypothesized that the thermal burns by the bone cement and the micromovement cause the appearance of these lucent zones, and that the gradually progressing lucent zones initiate tumor recurrence. Four patients in the present study exhibited evenly distributed lucent zones, with hardened edges, around the bone cement during the first 4–13 months (mean, 8 months) of follow-up. These zones were speculated to be related to the thermal burns of bone cement; however, further follow-up indicated that these lucent zones demonstrated no further progression.

GCT often involves the subchondral bone and is sometimes situated near to the articular cartilage. Treatment with bone cement, thermal burns or micromovement theoretically causes cartilage degeneration and fracture, leading to the occurrence of osteoarthritis. Fraquet *et al*(9) treated 30 patients with long bone GCT by tumor curettage and bone cement filling, 73% of which exhibited GTC close to the articular cartilage. Following an average of 6.4 years’ follow-up, only two patients manifested mild joint degeneration. von Steyern *et al*(11) treated nine patients with GCT that were close to the knee joint with tumor curettage and bone cement filling. The distances between bone cement and articular cartilage were 0–3.5 mm (mean, 1.0 mm), of which three were 0 mm. During the 6–16 years of follow-up, only one case exhibited narrowness of the medial joint space in the postoperative weight-bearing X-ray. Although MR confirmed the existence of articular cartilage, delayed gadolinium-enhanced MR scan indicated articular cartilage injury. The authors stressed that the preoperative articular cartilage must be continuous, and noted that the hardness of subchondral filler is an important factor affecting the degeneration of articular cartilage. Previous animal experiments have confirmed that replacement of subchondral bone with bone cement does not reduce its strength (10). In addition, the nutrition of articular cartilage mainly depends on synovium and is less dependent on blood supply; therefore, the effect of bone cement filling on articular cartilage is not as serious as theoretically predicted. In this study, none of the 16 patients manifested clinical symptoms of osteoarthritis; however, due to the relatively short follow-up time, further follow-up observations are required.

Although there are few studies regarding the necessity and timing of internal fixation after bone cement filling, we propose that the internal fixation is necessary. Fraquet *et al*(9) indicated that without internal fixation, the stress-induced bone cement loosening would cause further resorption of the surrounding bone and pathologic fractures of the subchondral bone, leading to the bead effect of bone cement. Fixation is capable of locking the bone cement and bone cladding into one, hence preventing the occurrence of loosening. The authors treated 16 out of 30 cases of GCT of the long bone with internal fixation, and there was no bead effect observed with the bone cement. The current, commonly used internal fixation methods include fixations with steel plate or medullary cavity-implanted multiple Steinmann pins or cross screws. Toy *et al*(12) conducted *in vitro* biomechanical studies and revealed that bone cement filling of the distal femoral bone defect, accompanied by internal fixation using cross screws, generated significantly better biomechanical strength than that of simple bone cement filling, or bone cement filling in combination with fixation using intramedullary Steinmann pins. Uglialoro *et al*(13) demonstrated that the biomechanical strength of bone cement filling of the distal femoral defect, accompanied by internal steel plate fixation, is superior to that of bone cement filling in combination with fixations using intramedullary Steinmann pins or cross screws.

Bisphosphonate drugs have significant therapeutic effects on bone-metastasized tumors and osteoclast-mediated bone destructions, such as in GCT of the bone. Clinical studies have been conducted with regard to treating GCT with bisphosphonates as an adjuvant therapy. Tse *et al*(15) performed a retrospective controlled study of 44 cases of GCT patients. Of the 44 cases, 24 patients received two courses of preoperative and three courses of postoperative intravenous infusions of pamidronate disodium or zoledronic acid, followed by three months of oral administration of clodronate disodium. In the follow-up of 48–115 months, the recurrence rates in drug treatment and control groups were 4.2% (1/24) and 30% (6/20), respectively. However, due to the small experimental sample size and the fact that the follow-up was short-term (as well as many other influential factors), the ability of bisphosphonates to reduce the recurrence rate of GCT remains inconclusive. In this study, all patients received oral alendronate sodium tablets (10 mg/day) for two years with a one month interval between each two months of oral administration. The main side effects of alendronate sodium are acid reflux, heartburn and abdominal pain, among other gastrointestinal symptoms. The patients in this study manifested only low fever, acid reflux, heartburn and other mild adverse reactions, which disappeared after symptomatic treatment, suggesting a good safety of the drug.

In summary, we have applied intralesional aggressive curettage, bone cement filling and plate internal fixation in 16 GCT cases. This was followed by postoperative oral administration of bisphosphonate drugs (alendronate sodium), and no recurrence has been observed since. For clinical practice, our method has advantages including being easy to perform, ideal recovery of the limb function, low rate of short-term recurrence and acceptance by patients. The median follow-up time of this study was 28 months; therefore, a greater number of cases and longer follow-up times need to be investigated in the future.
